# Autosomal Recessive *NRL* Mutations in Patients with Enhanced S-Cone Syndrome

**DOI:** 10.3390/genes9020068

**Published:** 2018-01-30

**Authors:** Karin W. Littink, Patricia T. Y. Stappers, Frans C. C. Riemslag, Herman E. Talsma, Maria M. van Genderen, Frans P. M. Cremers, Rob W. J. Collin, L. Ingeborgh van den Born

**Affiliations:** 1 The Rotterdam Eye Hospital, 3011 BH Rotterdam, The Netherlands; k.littink@oogziekenhuis.nl (K.W.L.); p.stappers@oogziekenhuis.nl (P.T.Y.S.); FRiemslag@gmail.com (F.C.C.R.); HTalsma@bartimeus.nl (H.E.T.); 2 Bartiméus Center for Complex Visual Disorders, 3703 AJ Zeist, The Netherlands; mvgenderen@bartimeus.nl; 3 Department of Human Genetics, Radboud University Medical Center, 6525 GA Nijmegen, The Netherlands; Frans.Cremers@radboudumc.nl (F.P.M.C.); Rob.Collin@radboudumc.nl (R.W.J.C.); 4 Donders Institute for Brain, Cognition and Behaviour, Radboud University Medical Center, 6525 GA Nijmegen, The Netherlands

**Keywords:** enhanced S-cone syndrome, autosomal recessive *NRL*, hereditary retinal dystrophy, S-cone-specific ERG

## Abstract

Enhanced S-cone syndrome (ESCS) is mainly associated with mutations in the *NR2E3* gene. However, rare mutations in the *NRL* gene have been reported in patients with ESCS. We report on an ESCS phenotype in additional patients with autosomal recessive *NRL* (ar*NRL*) mutations. Three Moroccan patients of two different families with ar*NRL* mutations were enrolled in this study. The mutation in the DNA of one patient, from a consanguineous marriage, was detected by homozygosity mapping. The mutation in the DNA of two siblings from a second family was detected in a targeted next-generation sequencing project. Full ophthalmic examination was performed, including best-corrected visual acuity, slit-lamp biomicroscopy, funduscopy, Goldmann kinetic perimetry, optical coherence tomography, fundus autofluorescence, and extended electroretinography including an amber stimulus on a blue background and a blue stimulus on an amber background. One patient carried a homozygous missense mutation (c.508C>A; p.Arg170Ser) in the *NRL* gene, whereas the same mutation was identified heterozygously in the two siblings of a second family, in combination with a one base-pair deletion (c.654del; p.Cys219Valfs*4) on the other allele. All patients had reduced visual acuity and showed a typical clumped pigmentary retinal degeneration (CPRD). Foveal schisis-like changes were observed in the oldest patient. An electroretinogram (ERG) under dark-adapted conditions showed absent responses for low stimulus strengths and reduced responses for high stimulus strengths, with constant b-wave latencies despite increasing stimulus strength. A relatively high amplitude was detected with a blue stimulus on an amber background, while an amber stimulus on a blue background showed reduced responses. The ar*NRL* mutations cause a phenotype with typical CPRD. This phenotype has previously been described in patients with ESCS caused by *NR2E3* mutations, and rarely by *NRL* mutations. Based on our findings in ERG testing, we conclude that S-cone function is enhanced in our patients in a similar manner as in patients with *NR2E3*-associated ESCS, confirming previous reports of *NRL* as a second gene to cause ESCS.

## 1. Introduction

Enhanced S-cone syndrome (ESCS) is a rare autosomal recessive retinal degeneration characterized by an increased number of cones in the retina, of which the majority expresses S-cone opsins [1]. The typical features that distinguish ESCS from other retinal dystrophies are the pathognomonic responses on electroretinogram (ERG) measurements, consisting of an absence of rod response on low-intensity dark-adapted stimulus, and a simplified and delayed waveform at high-intensity dark-adapted stimulus [2,3,4]. On ophthalmoscopy, ESCS shows characteristic clumped pigment deposits and atrophy in the midperipheral fundus at the level of the retinal pigment epithelium (RPE), frequently associated with macular schisis, and dot-like lesions. Symptoms include night blindness and variable loss of visual acuity and visual field [3,4,5,6].

ESCS is almost exclusively caused by mutations in nuclear receptor subfamily 2, group E, member 3 (*NR2E3*; MIM # 604485). *NR2E3* encodes a ligand-dependent transcription factor that plays a role in rod differentiation [7], and suppresses short (S) wavelength cone opsins by interacting either directly or indirectly with other transcription factors, including cone-rod homeobox (CRX) and NRL [8,9,10]. *NRL* encodes a basic-motif leucine zipper (b-ZIP) DNA binding protein that is expressed in postmitotic rod photoreceptors and in the pineal gland. NRL interacts with CRX, NR2E3 and other transcription factors, and synergistically regulates the activity of rod-specific genes, thus guiding a postmitotic photoreceptor into the rod lineage [11,12,13]. More specifically, NRL binds directly to the promoter of *NR2E3* and thus acts upstream of this transcription factor [14]. In the retina of the *Nrl^-/-^* mouse, *Nr2e3* is also not expressed, and rods are transformed to functional S-cones [15]. Together, this made *NRL* a strong candidate to be mutated in patients with ESCS. 

Autosomal dominant missense mutations in *NRL* have been reported, to underlie retinitis pigmentosa (RP) [16,17]. Only eight patients, carrying six different mutations, have been described carrying autosomal recessive (ar) mutations in *NRL* [18,19,20,21,22]. Nishiguchi et al. reported two siblings with compound heterozygous *NRL* mutations: a loss-of-function mutation (c.224_225insC, p.Ala76Glyfs*18) and a missense mutation (c.479T>C, p.Leu160Pro). Their fundus showed the typical clumped retinal deposits. Relatively preserved visual field on blue-on-yellow perimetry, performed in one of the siblings, suggested that S-cone function was preserved in a similar manner as found in patients with ESCS. ERG recordings in this patient demonstrated a considerable reduction of rod and cone amplitudes, which precluded an evaluation of S-cone function by specific ERG testing using stimuli with different colors [18]. More recently, Newman et al. described two unrelated patients affected by oculopharyngeal muscular dystrophy (OPMD) accompanied by night blindness and reduced visual acuity, not commonly associated with OPMD. Mutation analysis in these patients revealed the same homozygous loss-of-function mutation (c.91C>T; p.Arg31*) in *NRL* in both patients. Extensive ERG recording in those patients revealed a ESCS-like phenotype characterized by dominance of the short-wavelength-sensitive responses with no detectable rod function [19]. Four other patients carrying ar*NRL* mutations have been detected by Beryozkin et al. and our own group, using different methods to identify sequence variants in large groups of presumed ar retinitis pigmentosa (RP) patients (high resolution genome-wide homozygosity mapping, targeted next-generation sequencing, and whole exome sequencing, respectively) [20,21,22]. Clinical re-examination of one patient showed a clumped pigmentary retinal dystrophy (CPRD) phenotype [20], whereas the phenotype of the other three patients was not specified other than arRP [21,22]. 

In this study, we describe in detail the clinical phenotype of three ar*NRL* patients carrying autosomal recessive *NRL* mutations [20,21]. ERG testing, using the extended ISCEV protocol, in these patients showed absent rod responses, and the characteristic simplified and delayed waveform, typical in ESCS patients [2,3,4]. Additional light-adapted blue stimuli on an amber background and amber stimuli on a blue background, showed that the simplified waveform was solely explained by an enhanced S-cone response, confirming that autosomal recessive mutations in *NRL* indeed cause ESCS.

## 2. Materials and Methods

### 2.1. Genetic Analysis

This study conformed to the tenets of the Declaration of Helsinki (Seoul 2008), and was approved by the Ethics Committee of the Erasmus Medical Centre (ABR-NL34152.078.10), Rotterdam, The Netherlands. Informed consent was obtained from the participants before EDTA-anticoagulated venous blood was collected. Genomic DNA was isolated using standard procedures [23] at the Department of Human Genetics, Radboud University Nijmegen Medical Centre, Nijmegen, The Netherlands.

Three Moroccan patients of two different families were enrolled in this study. DNA of one patient (patient 32666), from a consanguineous marriage, was analyzed by homozygosity mapping [20]. DNA of two siblings of another family (patient 32594 and 32595) was included in a targeted next-generation sequencing project [21]. After identification of the genetic defect, segregation analysis was performed in both families by PCR amplification, followed by direct and indirect sequence analysis of the *NRL* gene. Details of molecular studies have been published [20,21]. Both families were from the Al Hoceima region in the northern part of Morocco, but were unrelated as far as known.

### 2.2. Clinical Evaluation

Clinical data were reviewed retrospectively, and subsequently prospective ophthalmic examination was performed in all patients, including best-corrected visual acuity (BCVA; Snellen chart), slit-lamp biomicroscopy, funduscopy, Goldmann kinetic perimetry (targets V-4e, and I-4e to I-1e), optical coherence tomography (OCT) and fundus autofluorescence (FAF). Fundus photographs were taken in 4 quadrants. Spectral-domain optical coherence tomography (SD-OCT) and fundus autofluorescence (FAF; Heidelberg Spectralis HRA+OCT, Heidelberg Engineering, Heidelberg, Germany) imaging were performed as described elsewhere [24].

ERG was performed according to the extended protocol for full-field ERG of the International Society for Clinical Electrophysiology of Vision (ISCEV) [25]. In addition, we performed ERG testing using an amber stimulus (2.0 cd·s·m^−2^) on a blue background (15 cd·m^−2^) and a blue stimulus (2.0 cd·s·m^−2^) on an amber background (15 cd·m^−2^) in two patients. This included a one-minute adaptation to an amber background to deactivate the L- and M-cones, followed by a series of 10 blue light-flashes (0.5 Hz) to stimulate the S-cones. This process was repeated, but instead patients followed a one-minute adaptation to a blue background to deactivate the S-cones and amber light-flashes were delivered to stimulate the L- and M-cones. All ERG responses were recorded with the Espion ColourDome and console (Diagnosys, LLC, Cambridge, UK) using Dawson Trick Litzkow (DTL)-fibre electrodes. Reference values were based on right eyes from 106 controls (0–70 years) that underwent ERG for diagnostic workup but in whom subsequently a retinal disorder was excluded. R_BvsA_ = 1.5 ± 0.4 (mean (m) + standard deviation (SD); *N* = 106). *R^2^* = 0.70 ± 0.14. Values within the limits of m ± 2 SDs of the controls were defined as normal.

Colour vision was performed in patient 32666 using the Panel D15, Ishihara and Hardy Rand Rittler (HRR) test plates. 

## 3. Results

In three patients (32666, 32595, 32594) from two unrelated Moroccan families, biallelic recessive *NRL* mutations have been identified (Table 1) [20,21].

**Figure 1:**
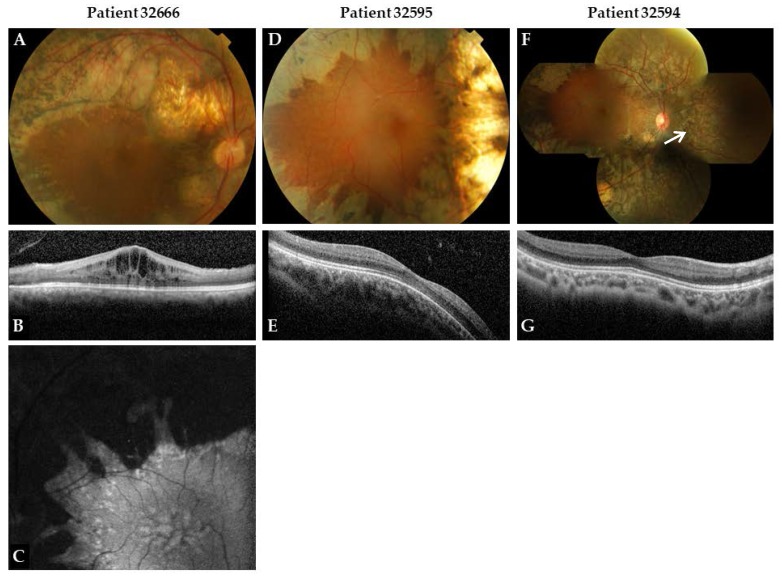
Fundus photographs and optical coherence tomography (OCT) of the right eye of patient 32666 at age 29, patient 32595 at age 8 and patient 32594 at age 7. Fundus autofluorescence (FAF) of the left eye of patient 32666 at age 29. All fundus images (**A**,**D**,**F**) show a pink optic disk, relatively normal arterioles (**D**,**F**) or slightly attenuated arterioles (**A**). All show midperipheral pronounced retinal pigment epithelium (RPE) atrophy with nummular pigmentations, and a preserved macular region. Subtle yellow dots are visible along the border of atrophic and preserved RPE. A vertical line was observed in patient 32594 ((**F**), arrow), suggesting subretinal fibrosis. OCT images show cystoid maculopathy with intact ellipsoid zone (EZ) (**B**), and a normal architecture of the retinal layers (**E**,**G**). FAF image of the left eye of patient 32666 (**C**) shows pronounced hypoautofluorescence outside the vascular arcade with central hyperfluorescent spots, and spoke-like relative increased autofluorescence in the macula.

Patient 32666 was first seen at The Rotterdam Eye Hospital (Rotterdam, The Netherlands) at the age of 10 with night blindness, a divergent strabismus and uncorrected hyperopia (+4.75/+6.50 D spherical equivalent (SE)). At first visit his best-corrected visual acuity (BCVA) was 20/100 in both eyes (Table 1). Visual acuity slowly deteriorated until 26 years of age (20/200 both eyes; Table 1, Proband 32666), and dense posterior subcapsular cataracts developed. After cataract extraction at the age of 27, visual acuity was 20/125 in both eyes. Typical fundus abnormalities were observed form the start and were slowly progressive through the years. These consisted of pink optic discs, slightly attenuated arterioles, and preserved RPE at the macular region but with pronounced cysts (retinoschisis) (Figure 1A–C). In the midperiphery, nummular pigmentations at the level of the RPE with pronounced RPE atrophy were observed, together with a few torpedo-like lesions along the vascular arcades consisting of central chorioretinal atrophy with a hyperpigmented border. The RPE nasally to the disc was also affected. Centrally to the degenerative changes, tiny hypopigmented changes were noted pointing towards the macula, giving the impression of a stellate type of degeneration.

OCT of the macula region showed cystoid changes in both eyes (Figure 1B) with a preserved ellipsoid zone (EZ). The cystoid maculopathy was unresponsive to treatment with acetazolamide 250 mg *b.i.d.* Fundus autofluorescence (FAF) showed pronounced hypoautofluorescense outside the vascular arcade with central hyperautofluorescent spots along the border of the hypoautofluorescent area, and spoke-like relative increased autofluorescence in the macula (Figure 1C). Goldmann visual field was moderately constricted with midperipheral and central sensitivity loss, and revealed slow deterioration over 19 years. 

Electroretinography (ERG) at the age of 10 under dark-adapted conditions showed high responses, with an abnormal shape with 1 Hz flash interval, and minimal responses with an interval of 6 Hz. The most recent ERG was performed at 29 years of age, and did not reveal any responses under dark-adapted and light-adapted conditions. A 30-Hz flicker stimulation resulted in low responses (16 µV) with increased latencies. Light-adapted blue flash on an amber background showed a slow small response, whereas the amber flash on a blue background showed no response. Red–green color vision defect was found in the Panel D15, Ishihara and HRR tests. 

Patients 32595 and 32594 both presented at the age of 2 years with a convergent strabismus and hyperopia (32595: +1.75/+3.00 D SE. 32594: +2.50/+3.75 D SE), horizontal nystagmus, and photophobia. Funduscopy at first visit showed a clumped pigmentary retinopathy with pink optic discs, normal arterioles, and a preserved macular region without cysts. As in subject 32666, there was pronounced RPE atrophy with nummular pigmentations in the midperiphery. The atrophy extended beyond the vascular arcades and nerve head, and had the configuration of torpedo-like lesions without hyperpigmented borders in the oldest sibling. In the youngest sibling, the atrophy was almost confluent with hyperpigmented borders (Figure 1D,F). Centrally and peripherally to the atrophy we observed yellow dot-like lesions and RPE changes. In the youngest sibling, we also observed a vertical line temporally of the optic nerve head in the right eye suggesting subretinal fibrosis, but this was not confirmed by OCT. 

In the oldest sibling (32595), visual acuity seemed stable so far; BCVA was 20/40 (+3.0/+3.0 D SE) in both eyes, from 5 until 13 years of age. In the youngest sibling (32594), BCVA changed over time from 20/40 in both eyes at the age of 6 years, to 20/32 (+1.50 D SE) and 20/63 (+3.25 D SE) at age 11 (Table 1). Slit-lamp biomicroscopy revealed the presence of vitreous cells in both patients. No lens opacities were noted. 

OCT of the macular area showed a normal architecture of the retinal cell-layers in patients 32594 and 32595 (Figure 1E,G). FAF could not be performed because of severe photophobia and nystagmus. Goldmann visual field showed midperipheral and central sensitivity loss, and was moderately constricted in both siblings.

**Figure 2:**
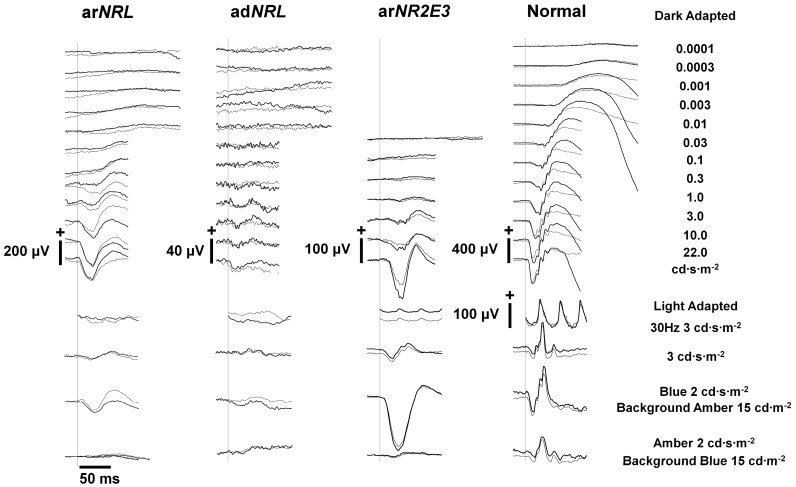
Electroretinography of subject 32594 with ar*NRL* mutations. ERG results of proband 32594 with ar*NRL* mutation c.508C>A and c.654del. The dark-adapted ERG shows absent rod responses for low stimulus strengths and reduced combined rod-cone responses for high stimulus strengths, with constant a-wave latencies despite increasing stimulus strength. The light-adapted ERG shows reduced and delayed responses to a 3 cd·s·m^−2^ stimulus (cone response). Light-adapted responses show a relatively high amplitude for a blue stimulus on an amber background, and reduced responses for an amber stimulus on a blue background. Also shown are ERG results of age-matched individuals with autosomal dominant *NRL* mutation c.152C>A (ad*NRL*) with retinitis pigmentosa, homozygous *NR2E3* mutation c.119-2A>C (ar*NR2E3*) with ESCS, and a normal individual.

Standard ISCEV ERG examination was performed in both patients, at respectively 2 and 4 years of age (patient 32595) and 2 and 3 years of age (patient 32594), and seemed non-recordable probably due to the skin electrodes. More recent extended dark-adapted ERGs (respectively at 8 and 7 years of age) measured with DTL electrodes showed comparable results in both patients; absent responses for low stimulus strengths and reduced responses for high stimulus strengths, with constant a-wave latencies despite increasing stimulus strength. Light-adapted ERG responses showed a 10-fold reduction compared to normal individuals, and displayed a simple waveform, with a constant a-wave latency as well. Relatively high amplitudes were detected with a blue stimulus on an amber background, while an amber stimulus on a blue background showed reduced responses (Figure 2).

## 4. Discussion

We studied three patients of two different Moroccan families with ar*NRL* mutations and confirmed by ERG measurements that autosomal recessive *NRL* mutations can cause ESCS. 

Ar*NRL* mutations seem very rare; only eight patients from six unrelated families with autosomal recessive mutations in *NRL* have been described. An ESCS phenotype was suggested by Nishigushi et al. based on chromatic Humphrey static perimetry in the two siblings [18]. Newman et al. confirmed the predominance of S-cones in two patients, who are also affected by oculopharyngeal muscular dystrophy, by S-cone-specific ERG measurements, showing the typical delayed waveform to white light stimulus which is similar in dark-adapted as well as light-adapted responses [19]. Beside these four patients, ESCS was thus far exclusively associated with autosomal recessive mutations in *NR2E3*, explaining up to 93% of cases [5,26]. 

*NRL* has been a likely candidate gene for ESCS, since the phenotype of the *Nrl^-/-^* mouse showed a complete loss of rod function and super-normal cone function, dominated by S-cones [15]. ERG responses of the *Nrl^-/-^* mouse retina showed complete absence of rod responses. Instead, a supernormal S-cone function was demonstrated by the six-times-enlarged amplitudes in response to S-cone specific stimuli (400 nm). Dominance of S-cone function was further demonstrated by morphological, immunohistochemical and gene-expression analysis, which showed the presence of S-cone immunoreactivity and an increase in S-cone-specific gene expression, in otherwise morphologically indistinct outer segment that were neither rods, nor cones. No rhodopsin immunoreactivity was detected. Phenotypic similarities between the *Nrl^-/-^* mouse phenotype and ESCS made *NRL* an excellent candidate gene for ESCS. However, screening of the *NRL* gene in several cohorts of ESCS patients (a total of 44 ESCS patients, and 749 patients with different types of inherited retinal dystrophies) [18,26,27] revealed only two siblings carrying compound heterozygous mutations in *NRL* (p.L75fs and p.L160P) [18], indicating that autosomal recessive mutations in *NRL* are a rare cause of ESCS) [18,19,20,21].

Clinical examination of our three ar*NRL* patients showed a relatively homogeneous early-onset phenotype, encompassing reduced best-corrected visual acuity, nystagmus, hyperopia, constricted visual fields and pronounced fundus abnormalities at the age of diagnosis. The course of the disease was slowly progressive, concluded by repeated ERG measurements and visual field examinations, whereas the visual acuity seemed relatively stable thus far. In patient 32666, visual acuity was severely reduced at diagnosis (20/100 at age 10), maybe as the result of the cystoid maculopathy. In patients 32594 and 32595, who did not show signs of foveal schisis, visual acuity was relatively better (20/40 at age 13, and 20/32 (best eye) at age 11). Visual acuity in the other ar*NRL* patients varied between 20/40 in a 51-year-old patient to 20/480 in a 35-year-old [18,19]. No cystic changes were described in these patients. Funduscopic features of our patients were similar to four of the previously reported patients, encompassing a CPRD [18,19].

We recorded typical S-cone-dominated ERG responses in two of our patients: extended ISCEV ERG recordings showed the pathognomonic ERG responses typical for ESCS consisting of undetectable rod response to a low-intensity dark-adapted stimulus, a simplified and delayed waveform at high-intensity dark-adapted stimulus under dark-adapted conditions, and similar waveforms of the combined rod-cone and cone responses [2,5]. The simplified and similar waveforms result from the dominant contribution to the responses of the S-cones. In two of our patients, a blue stimulus after adapting to amber light showed a response comparable to the high-intensity dark-adapted stimuli, while amber stimulus following adaptation to blue light showed an almost undetectable response. The wave forms were highly similar to the ERG recordings of an age-matched ESCS subject carrying *NR2E3* mutations. The S-cone responses of the two ar*NRL* patients described by Newman et al. showed slightly different responses with super large a-waves and a relatively smaller b-wave, suggesting a negative wave form. They compared these findings with the wave forms of one ESCS patient, and concluded that the ar*NRL* responses were ESCS-like, but not identical [19]. However, they used a different protocol with a stronger flash than in our patients. These observations illustrate that differences in protocols might lead to other conclusions. One should also keep in mind that interpatient variability and progression of the disease will also influence ERG outcomes [5]. 

Besides the ERG, there are more clinical similarities between ESCS caused by *NRL* and *NR2E3* mutations, like age of onset in childhood, nyctalopia, normal-to-reduced visual acuity, hyperopia, and constricted visual fields, a variable degree of divergent or convergent strabismus, and a slowly progressive nature of the disease [4,5,26,28]. Subcapsular cataract and nystagmus as in one and two of our patients respectively is not frequently reported in ESCS patients carrying *NR2E3* mutations [5,28]. Despite the reduction in L- and M-cone sensitivity, color vision in ESCS patients assessed with standard tests is usually normal [29], but some deficits have been reported. Audo et al. reported on elevated protan and deutan thresholds as also observed in our eldest subject [5]. 

Fundus abnormalities in *NR2E3* patients may be highly variable, ranging from normal to CPRD with marked pigmentary changes [5,28,30]. Hull et al. reported on 9 children (7–15 years of age) with ESCS due to *NR2E3* mutations. The typical ESCS funduscopic features were present in only 6 of 9 patients at presentation. Evaluation of these patients suggested a sequence of changes in funduscopy, ranging from a normal fundus at presentation, followed by RPE mottling along the arcades, then the development of white dots, followed by deep nummular pigmentary deposition [30]. Audo et al., on the other hand, described patients with only subtle pigmentary changes up to the age of 72 [5]. The funduscopic changes observed in our three ar*NRL* patients in the elliptical ring near the vascular arcades seemed to be more extensive at a young age compared to the ESCS phenotype caused by *NR2E3*, but the number of described patients with ar*NRL* mutations is too low to draw any conclusions on severity of disease compared to patients with *NR2E3* mutations. The configuration of the chorioretinal atrophy along the vascular arcades in patient 32595, and the hyperpigmented border in patient 32666 resemble the description by Yzer et al. of torpedo-like lesions in ESCS patients with *NR2E3* mutations, but in a more pronounced way [28]. 

A feature also frequently reported in *NR2E3*-associated ESCS is yellow dots in areas of relatively normal-appearing peripheral retina [28], within the posterior pole or in areas of marked pigmentary abnormalities [5]. Comparable yellow deposits were seen in our patients and in two patients described previously, and seemed to be associated with hyperautofluorescence on FAF. Audo et al. hypothesized that these yellow dots may be related to the white dots seen in *rd7* mice carrying homozygous *NR2E3* mutations that histologically correspond to pseudorosettes of dysplastic photoreceptors [5]. These pseudorosettes were observed in *Nrl^-/-^* mice as well, disappearing after 31 weeks, resulting in thinning of the outer nuclear layer [15]. Whether the yellow dots are the precursor of RPE atrophy and torpedo-like lesions is currently unknown, but Yzer et al. speculated that regions with abnormal photoreceptor and RPE approximation, possibly caused by retinal folds or pseudorosettes, as seen in mouse models and human donor retina, might lead to the round or oval regions of RPE cell loss [1,15,28]. Subretinal fibrosis in the parapapillary area, a feature described in the same paper, was suspected in our youngest subject.

Schisis-like abnormalities with or without cystoid changes are also considered to be a classic hallmark of ESCS and were observed in 12 out of 28 *NR2E3* patients as described in the papers by Audo et al. and Hull et al. [5,30], and in one of our ar*NRL* patients. Five of them (4 *NR2E3*, 1 ar*NRL*) were treated with oral acetazolamide, and none of them responded to this treatment. Audo et al. hypothesized that the cystoid changes were more likely secondary to foveal schisis, since no leakage on fundus fluorescence imaging was noted [15,28].

In conclusion, ERG testing with additional short wavelength specific stimuli has shown that S-cone function is preserved in ar*NRL* patients in a similar manner as in patients with ESCS due to mutations in *NR2E3*, confirming that *NRL* is the second gene to cause ESCS.

## Figures and Tables

**Table 1:** Overview of clinical and genetic findings in patients with autosomal recessive *NRL* mutations.

Proband	Gender	Mutation (cDNA)	Mutation (Protein)	Age at Diagnosis (y)	Age at Last Examination (y)	BCVA RE, LE Refr. Error (SE)	Goldmann Perimetry	Ocular Features
32666	M	c.508C>A c.508C>A	p.Arg170Ser p.Arg170Ser	10	29	20/125, 20/125 +4.75/+6.50	Moderately constricted, midperipheral and central sensitivity loss	Divergent strabismus
32595	M	c.508C>A c.654del	p.Arg170Ser p.Cys219Valfs*4	2	13	20/40, 20/40 +3.0/+3.0	Midperipheral and central sensitivity loss	Convergent strabismus, nystagmus
32594	M	c.508C>A c.654del	p.Arg170Ser p.Cys219Valfs*4	2	11	20/32, 20/63 +1.5/+3.25	Moderately constricted, midperipheral and central sensitivity loss	Convergent strabismus, nystagmus

BCVA: best-corrected visual acuity; LE: left eye; RE: right eye; Refr. error: refractive error; SE: spherical equivalent; y: years.
