# Correction: Littink, K. W.; et al. Autosomal Recessive *NRL* Mutations in Patients with Enhanced S-Cone Syndrome. *Genes* 2018, *9*, 68

**DOI:** 10.3390/genes9030145

**Published:** 2018-03-07

**Authors:** Karin W. Littink, Patricia T.Y. Stappers, Frans C.C. Riemslag, Herman E. Talsma, Maria M. van Genderen, Frans P.M. Cremers, Rob W.J. Collin, L. Ingeborgh van den Born

**Affiliations:** 1 The Rotterdam Eye Hospital, 3011 BH Rotterdam, The Netherlands; k.littink@oogziekenhuis.nl (K.W.L.); p.stappers@oogziekenhuis.nl (P.T.Y.S.); FRiemslag@gmail.com (F.C.C.R.); HTalsma@bartimeus.nl (H.E.T.); 2 Bartiméus Center for Complex Visual Disorders, 3703 AJ Zeist, The Netherlands; mvgenderen@bartimeus.nl; 3 Department of Human Genetics, Radboud University Medical Center, 6525 GA Nijmegen, The Netherlands; Frans.Cremers@radboudumc.nl (F.P.M.C.); Rob.Collin@radboudumc.nl (R.W.J.C.); 4 Donders Institute for Brain, Cognition and Behaviour, Radboud University Medical Center, 6525 GA Nijmegen, The Netherlands

The authors wish to make the following correction to this paper [1]. Due to mislabeling, replace: 
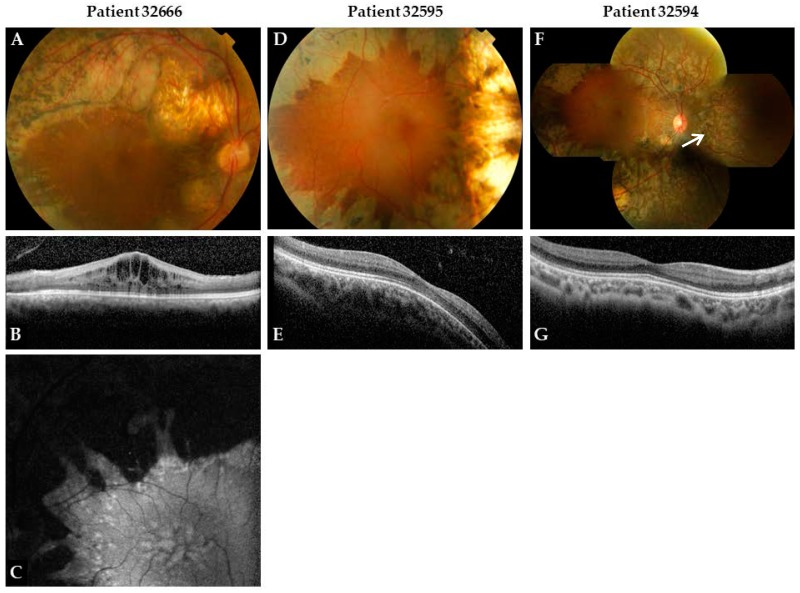
 with 
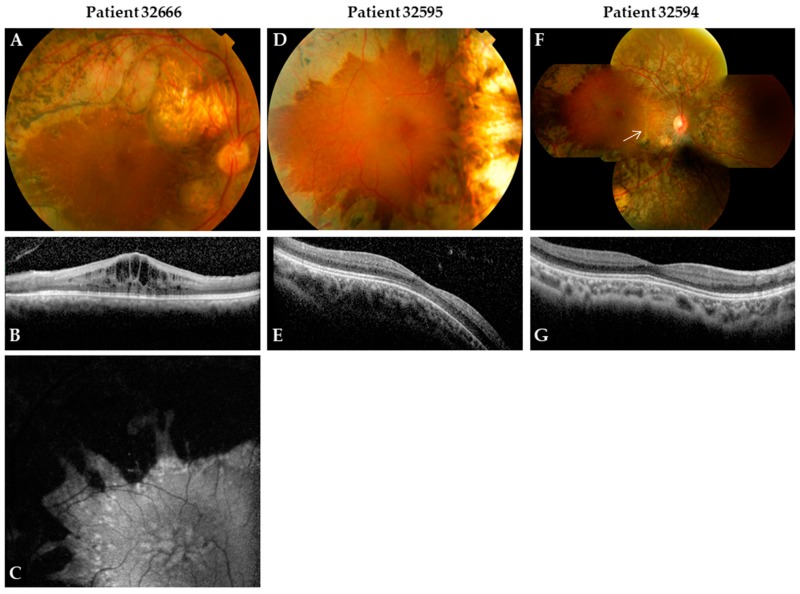


The authors would like to apologize for any inconvenience caused to the readers by these changes.
